# Supplementary material for: Glucocorticoid Receptor Binding Induces Rapid and Prolonged Large-Scale Chromatin Decompaction at Multiple Target Loci
Source: Cell Rep. 2017 Dec 12;21(11):3022–31. doi: 10.1016/j.celrep.2017.11.053 (PMC5745231; doi:10.1016/j.celrep.2017.11.053)
Supplement: Document S1. Figures S1–S5 [file mmc1.pdf]

Cell Reports, Volume *21*

Supplemental Information

Glucocorticoid Receptor Binding Induces Rapid and Prolonged Large-Scale Chromatin Decompaction at Multiple Target Loci

Alasdair W. Jubb, Shelagh Boyle, David A. Hume, and Wendy A. Bickmore

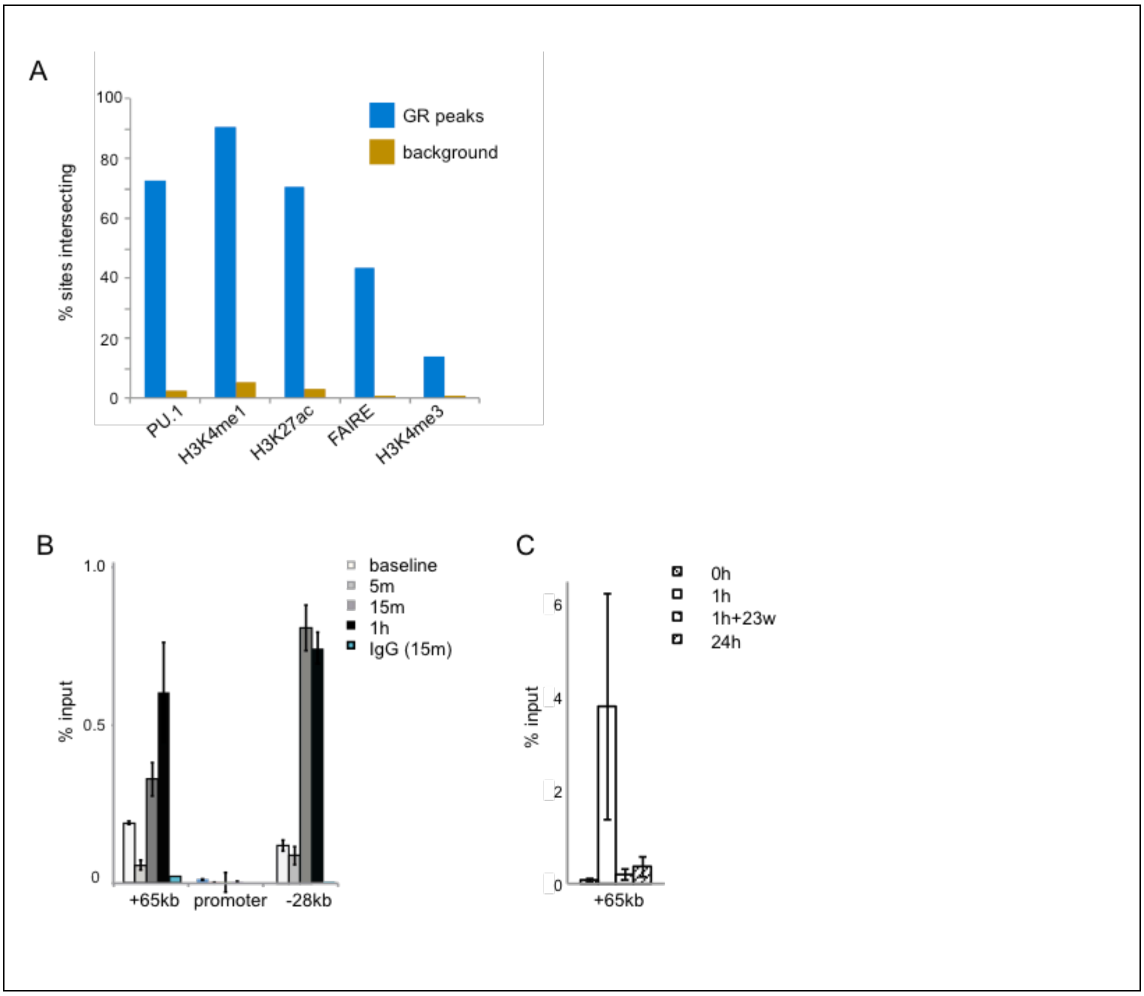

Figure S1. GR binding precedes detectable rises in *Fkbp5* mRNA and is lost by 24h. Related to Fig 1. (A) Percent of GR binding sites in mBMDM treated with Dex that overlap with the given features in un-treated mBMDMs. Comparison is between our previous GR data (Jubb et al., 2016) and published data for PU.1 binding, H3K4me1, H3K27ac, FAIRE-seq and H3K4me3 (Ostuni et al., 2013). Blue; data for GR peaks. Orange; data for a GC matched genome permuted background set of peaks. (B) Glucocorticoid receptor binding in Dex treated mBMDM measured by ChIP-qPCR for the downstream enhancer (+65kb), promoter and upstream enhancer (-28kb) of *Fkbp5*. Data is shown for a 4 point time series (baseline, 5min, 15min,1h) of treatment with 100nM dexamethasone. Normal IgG is also shown. Error bars are 2 x standard error of the mean (SEM) for 3 technical replicates. This data is a biological replicate of the data shown in Figure 1C. (C) Glucocorticoid receptor binding measured by ChIP-qPCR for the downstream enhancer (Fkbp5+65kb) following stimulation with 100nM dexamethasone at 1 hour and 24h with (1h+23w) and without (24h) washout of the ligand. Error bars are 2 x standard error of the mean (SEM) for 3 technical replicates. This is a biological replicate of the data in Figure 1G.

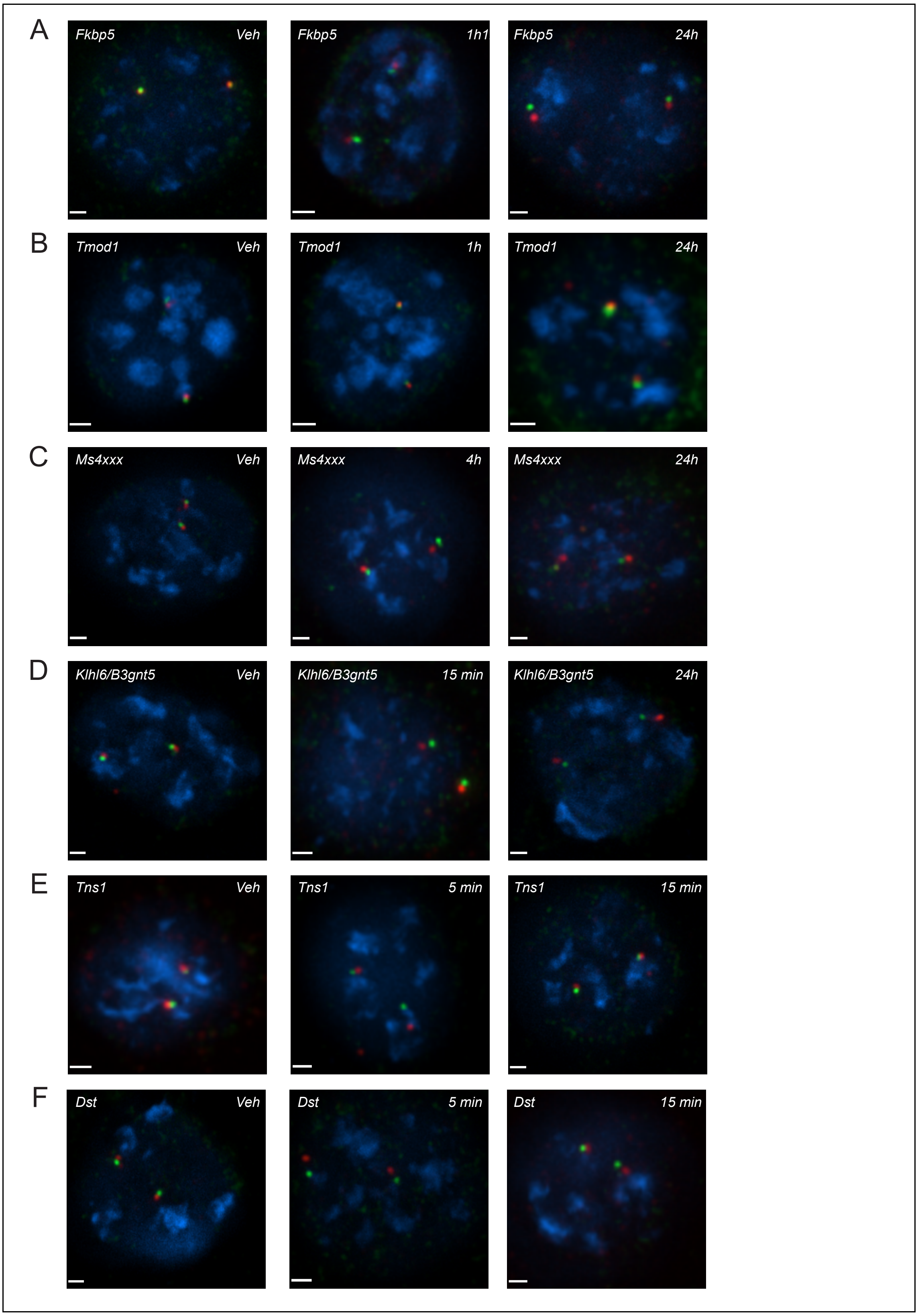

Figure S2. Illustrative DNA FISH images. Related to Figures 1, 2, 3 and 4. (A-F) Images of DNA FISH from each of the studied loci at the time points indicated. These images are presented for illustration purposes and represent merged 3D image stacks. Data collection was performed in 3D as described previously (Eskeland et al., 2010).

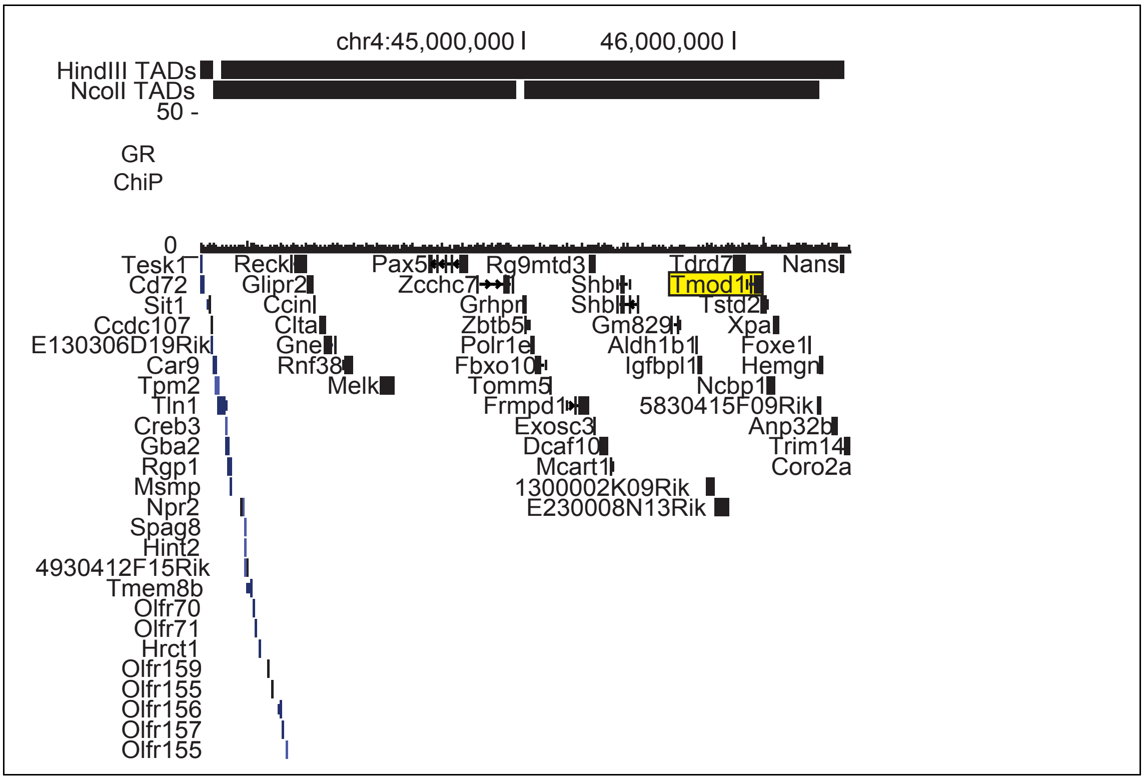

Figure S3. ***Tmod1* does not have GR bound in the same TAD. Related to Figure 2.** Very wide view from the UCSC genome browser of the *Tmod1* locus that shows no GR bound within either of the two sets of Topology Associated Domains (TADs) reported in (Dixon et al., 2012) using HindIII and NcoII restriction enzymes, TADs shown in black above locus.

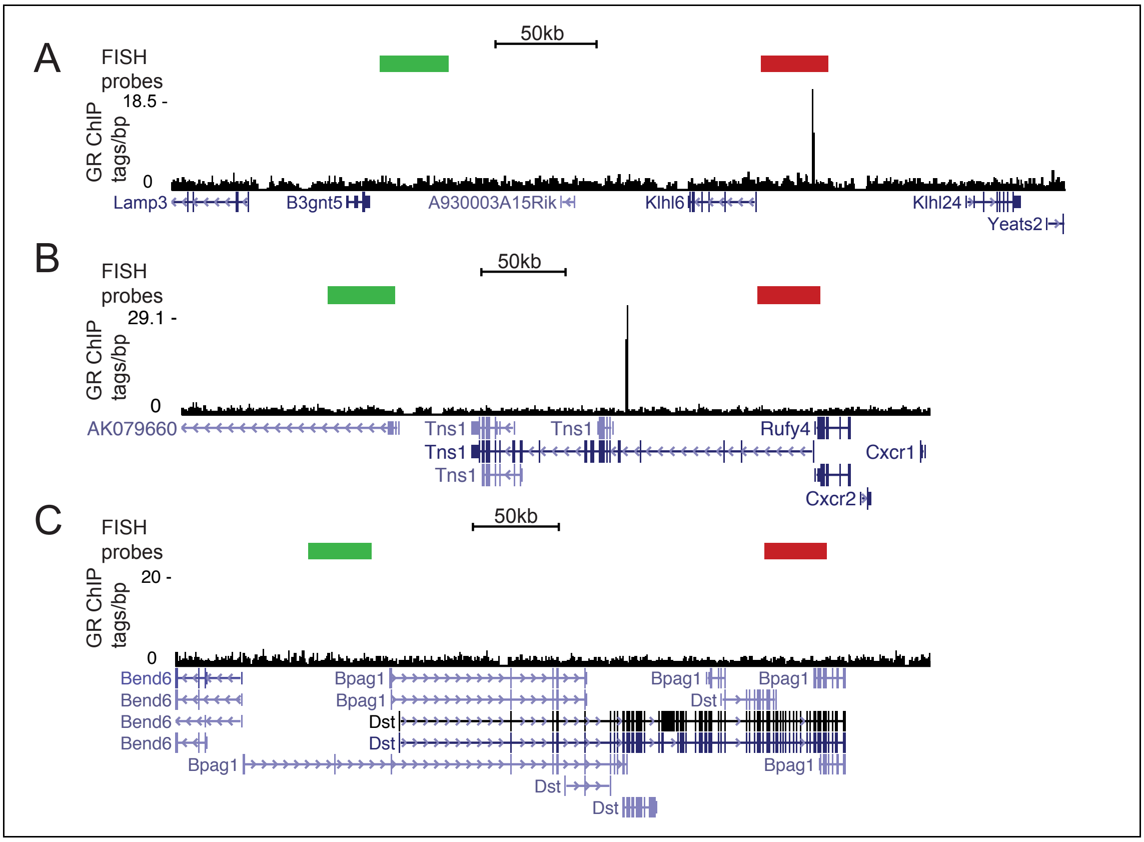

Figure S4. Locations of additional fosmid probes used for 3D DNA FISH. **Related to Figures 1, 2, 3 and 4**. (A) UCSC Genome browser image showing *Klhl6 / B3gnt5 / Klhl24* gene locus with ChIP-seq data (tags/bp) for GR binding from (Jubb et al. 2016). Red and green blocks show the positions of fosmid probes used for FISH analysis (mm9). (B & C) analogous images to (A) for *Tns1* and *Dst.*

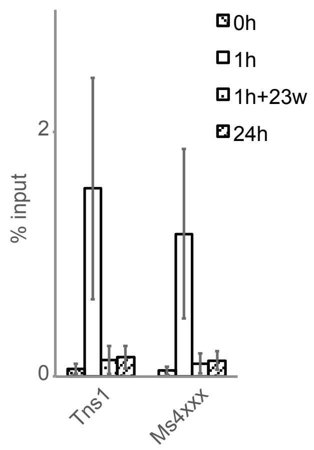

Figure S5. Glucocorticoid receptor binding is not present at two further target loci 24h after treatment. Related to Figure 4. Glucocorticoid receptor binding in Dex treated mBMDM measured by ChIP-qPCR at *Tns1* and *Ms4xxx* loci following stimulation with 100nM dexamethasone. Data is shown for 1 hour and 24h with (1h+23w) and without (24h) washout of the ligand. Error bars are 2 x standard error of the mean (SEM) for 3 technical replicates.
