# Supplementary material for: Glucocorticoid Receptor Binding Induces Rapid and Prolonged Large-Scale Chromatin Decompaction at Multiple Target Loci
Source: Cell Rep. 2017 Dec 12;21(11):3022–31. doi: 10.1016/j.celrep.2017.11.053 (PMC5745231; doi:10.1016/j.celrep.2017.11.053)
Supplement: Document S2. Article plus Supplemental Information [file mmc2.pdf]

Cell Reports

Report

Glucocorticoid Receptor Binding Induces Rapid and Prolonged Large-Scale Chromatin Decompaction at Multiple Target Loci

Graphical Abstract

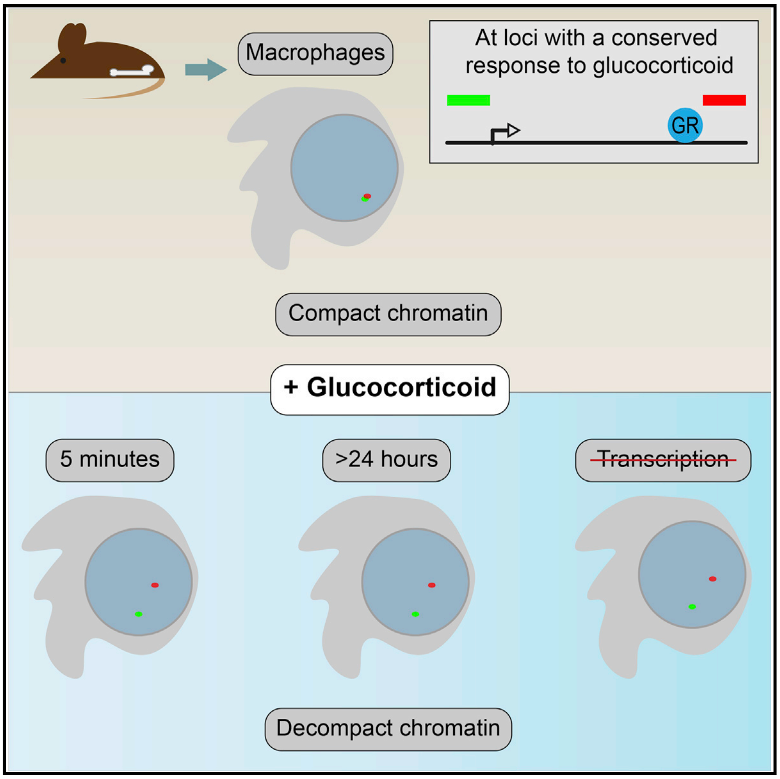

Highlights

- Glucocorticoids can induce rapid and persistent chromatin decompaction
- Transcription is not essential for chromatin decompaction
- Large-scale chromatin organization may modulate the glucocorticoid response

Authors

Alasdair W. Jubb, Shelagh Boyle, David A. Hume, Wendy A. Bickmore

Correspondence

david.hume@roslin.ed.ac.uk (D.A.H.), wendy.bickmore@igmm.ed.ac.uk (W.A.B.)

In Brief

Glucocorticoids are important anti-inflammatory therapeutics. Jubb at al. find that treating primary macrophages with glucocorticoid induces rapid and persistent chromatin reorganization at genes that respond in both mice and humans. Higher-order chromatin structure at these core loci may be involved in the glucocorticoid response and be relevant to resistance.

Data and Software Availability

GSE61881

Jubb et al., 2017, Cell Reports *21*, 3022–3031
December 12, 2017 © 2017 The Author(s).
https://doi.org/10.1016/j.celrep.2017.11.053

CellPress

OPEN ACCESS
CellPress

Cell Reports
Report

Glucocorticoid Receptor Binding Induces Rapid and Prolonged Large-Scale Chromatin Decompaction at Multiple Target Loci

Alasdair W. Jubb,[1,2,3] Shelagh Boyle,[1] David A. Hume,[2,4,*] and Wendy A. Bickmore[1,5,*]
[1]MRC Human Genetics Unit, Institute of Genetics and Molecular Medicine, The University of Edinburgh, Crewe Road, Edinburgh EH4 2XU, UK
[2]The Roslin Institute and Royal (Dick) School of Veterinary Studies, The University of Edinburgh, Easter Bush, Midlothian EH25 9RG, UK
[3]Department of Medicine, University of Cambridge, Box 93, Addenbrookes Hospital, Hills Road, Cambridge CB2 2QQ, UK
[4]Mater Research-University of Queensland, Translational Research Institute, 37 Kent Street, Woolloongabba, QLD 4102, Australia
[5]Lead Contact
*Correspondence: david.hume@roslin.ed.ac.uk (D.A.H.), wendy.bickmore@igmm.ed.ac.uk (W.A.B.)
https://doi.org/10.1016/j.celrep.2017.11.053

SUMMARY

Glucocorticoids act by binding to the glucocorticoid receptor (GR), which binds to specific motifs within enhancers of target genes to activate transcription. Previous studies have suggested that GRs can promote interactions between gene promoters and distal elements within target loci. In contrast, we demonstrate here that glucocorticoid addition to mouse bone-marrow-derived macrophages produces very rapid chromatin unfolding detectable by fluorescence *in situ* hybridization (FISH) at loci associated with GR binding. Rapid chromatin decompaction was generally not dependent on transcription at those loci that are known to be inducible in both mouse and human macrophages and was sustained for up to 5 days following ligand removal. Chromatin decompaction was not dependent upon persistent GR binding, which decayed fully after 24 hr. We suggest that sustained large-scale chromatin reorganization forms an important part of the response to glucocorticoid and might contribute to glucocorticoid sensitivity and resistance.

INTRODUCTION

Glucocorticoids (GCs) are clinically important metabolic hormones with powerful anti-inflammatory effects. They are among the most widely prescribed therapeutic agents, but cardio-metabolic side effects and resistance limit their therapeutic use. Furthermore, GC resistance emerges in many patients with chronic inflammatory disease, and the basis of the lack of efficacy in acute inflammation associated with sepsis is not well understood (Dendoncker and Libert 2017; Rodriguez et al., 2016). Dysregulation of endogenous GCs during severe illness has been linked to worse outcomes (Annane et al., 2000; Boonen et al., 2013).

GCs act by binding to the glucocorticoid receptor (GR) (*Nr3c1*), which is a ligand-activated transcription factor (TF). Macrophages express GR at high levels (Forrest et al., 2014; Lattin et al., 2008) and are major targets of the anti-inflammatory and therapeutic impacts of GC treatment (Jubb et al., 2016; Oh et al., 2017). Although there is evidence for direct trans-repression of pro-inflammatory genes by GCs (Uhlenhaut et al., 2013), the major mechanism of action appears to involve induction of feedback regulators, such as *Dusp1*, *IkBa*, *Tnfaip3*, and *Tsc22d3* (Jubb et al., 2016; Oh et al., 2017; Reddy et al., 2009; Vandevyver et al., 2012). As an inducer of gene expression, GR binds in a ligand-dependent manner to DNA at sites (enhancers) that may lie many tens of kilobases from the target gene (Lim et al., 2015; Jubb et al., 2016; Reddy et al., 2009; So et al., 2007; Stavreva et al., 2015; Uhlenhaut et al., 2013). GR binding is largely constrained to sites accessible to nuclease digestion and is associated with the nearby binding sites for pioneer TFs (Belikov et al., 2009; Biddie et al., 2011; Grøntved et al., 2013; Jubb et al., 2016; Starick et al., 2015). As a consequence, GR target genes can vary between tissue and cell types. In the case of macrophages, GR binding is strongly associated with binding sites for the macrophage lineage TF PU.1 (Jubb et al., 2016; Oh et al., 2017), and further binding sites become available upon inflammatory activation, associated with AP1 and RelA binding (Oh et al., 2017). The transcriptional response of macrophages to GCs varies markedly between humans and mice, associated with the gain and loss of GR binding sequence motifs (Jubb et al., 2016). GR binding can also initiate the formation of a more nuclease-sensitive local chromatin structure (Biddie et al., 2011; Burd and Archer 2013; John et al., 2008; Hakim et al., 2011; Stavreva et al., 2015). This may be transient, disappearing rapidly upon hormone withdrawal, or may persist well beyond the period of hormone treatment (Stavreva et al., 2015).

As well as altering local nucleosome structure, some nuclear hormone receptors, such as the estrogen receptor, can modulate chromatin structure at a scale sufficiently large to be detectable by light microscopy (Nye et al., 2002; Rafique et al., 2015). There have been few reports on GR actions at this level of chromosome structure. At a repetitive array of the mouse mammary tumor virus (MMTV) promoter, GR binding results in transcription-dependent visible chromatin decompaction over the course of a few hours (Müller et al., 2001). Conversely, 4C chromatin conformation capture assays of the GR-responsive *Lcn2* locus in a mammary adenocarcinoma cell line indicated that GR binding produced only a modest effect on long-range chromatin contacts captured with this approach (Hakim et al., 2011).

This is an open access article under the CC BY license (http://creativecommons.org/licenses/by/4.0/).

OPEN ACCESS
CellPress

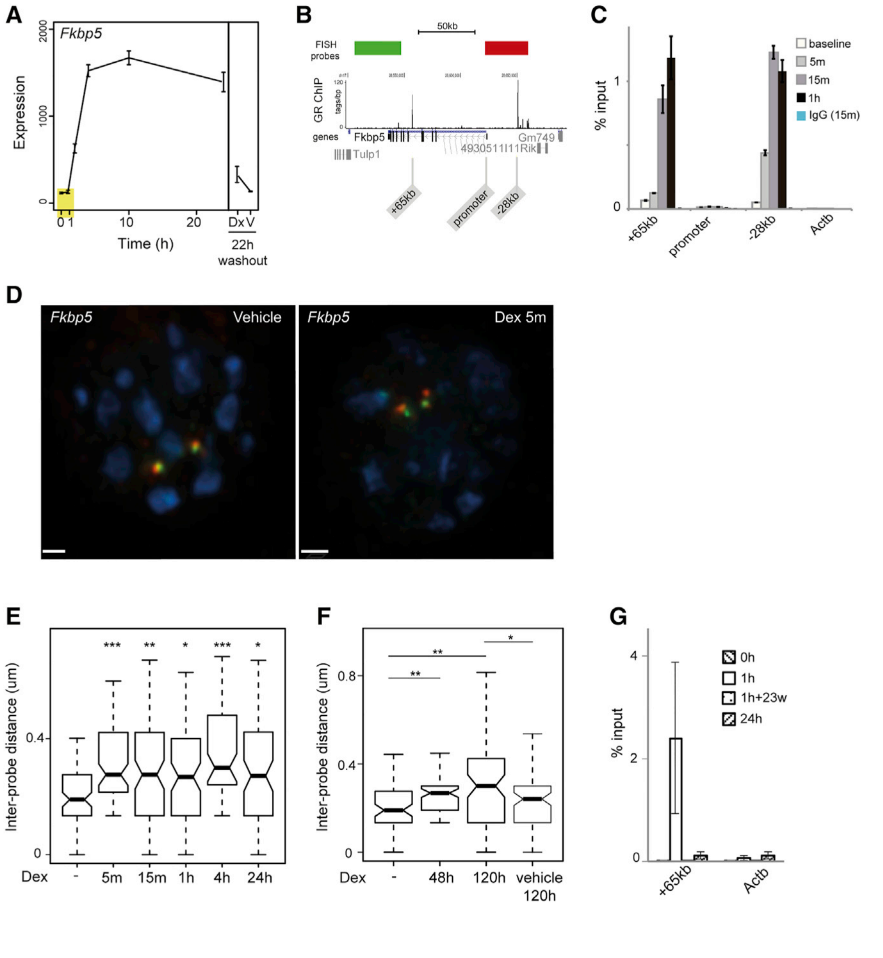

Figure 1. GC Causes Rapid and Prolonged Chromatin Decompaction at the *Fkbp5* Locus

(A) Expression data in response to 100 nM dexamethasone (Dex) for *Fkbp5* at the time points indicated (Jubb et al., 2016). Dx, treated for 2 hr, then washout for 22 hr; V, vehicle treated and washout 22 hr. Yellow rectangle highlights the static level of stable mRNA by 1 hr. Expression is presented as the raw normalized intensity values from microarrays.

(B) Genome browser image showing *Fkbp5* locus with ChIP-seq data (tags/bp) for GR binding from Jubb et al. (2016). Red and green blocks show the positions of FISH probes. Genome map positions are from the mm9 assembly of the mouse genome. Positions of primers used for ChIP-qPCR are shown (+65 kb, promoter, −28 kb).

(C) Glucocorticoid receptor (GR) binding measured by ChIP-qPCR for the downstream enhancer (+65 kb), promoter and upstream enhancer (−28 kb) of *Fkbp5* and a control site in the *Actb* promoter. Data are shown for a four-point time series (baseline, 5 min, 15 min,1 hr) of treatment with 100 nM Dex. IgG control ChIP is also shown. Error bars are 2× SEM for three technical replicates (a further biological replicate is shown in Figure S1B).

(D) 3D DNA FISH images of nuclei from mBMDM treated with 100 nM Dex or vehicle control for 5 min using the probes indicated in (A). Scale bar represents 1 μm.

(E) Boxplots of inter-probe distances (μm) measured across the *Fkbp5* locus at the indicated times following treatment with vehicle or 100 nM Dex; n = 80 for each condition. Horizontal line, median; whiskers, 1.5× interquartile range; *p < 0.05, **p < 0.005, ***p < 0.0005, Wilcoxon rank sum.

(F) As for (E) but after culturing for prolonged periods after Dex washout.

(G) GR binding measured by ChIP-qPCR for the *Fkbp5* downstream enhancer (+65 kb) and *Actb* promoter following stimulation with 100 nM Dex at 1 and 24 hr with (1h+23w) and without (24h) washout of the ligand. Error bars are 2× SEM for three technical replicates (a further biological replicate is shown in Figure S1C).

We previously identified inducible sites of GR binding at likely enhancers of genes induced by GC in primary mouse and human macrophages (Jubb et al., 2016). Here, using fluorescence *in situ* hybridization (FISH), we demonstrate rapid, persistent, and visible chromatin decompaction—increases in inter-probe distances—at multiple GC-responsive loci in mouse bone-marrow-derived macrophages (mBMDMs) following dexamethasone treatment. We compare and contrast the behavior of loci in which the GC response is conserved in mice and humans with loci whose response to GC is specific to mouse macrophages. Our data provide insight into how GCs affect large-scale chromatin structure in macrophages and indicate that there may be a long-term "memory" of GR binding on chromatin.

RESULTS

GC Induces Rapid GR Binding at Enhancers at the *Fkbp5* Locus

We have previously generated genome-wide expression and GR binding data in primary mouse and human macrophages responding to 100 nM dexamethasone (Dex), a GR agonist (Jubb et al., 2016). These GR binding sites bear the hallmarks of enhancers, being enriched in PU.1 binding sites, and active enhancer histone marks in unstimulated mBMDMs (Ostuni et al., 2013) (Figure S1A). Many GC-inducible genes were regulated only in one species or the other, associated with gain and loss of GR motifs from the respective genome. Even for genes that were induced in both species, the precise site of GR binding was not always conserved. In this study, we first focused on *Fkbp5*, a GR co-chaperone and inducible feedback regulator of the GC response. Elevated Fkbp5 levels reduce the affinity of GR for the agonist and are associated with resistance (Denny et al., 2000; Jääskeläinen et al., 2011; Vandevyver et al., 2012; Zannas et al., 2016).

Fkbp5 is strongly induced by Dex in both mouse and human macrophages. The time course of *Fkbp5* mRNA induction in BMDMs is shown in Figure 1A. In human A549 lung carcinoma cells, GR binding sites have been described 34 kb 5′ and 87 kb 3′ of the *FKBP5* transcription start site (TSS) (Reddy et al., 2009). In human macrophages, we also identified two major GR binding sites in similar locations (Jubb et al., 2016). In mBMDMs, chromatin immunoprecipitation sequencing (ChIP-seq) revealed strong GR binding at a site 28 kb upstream of *Fkbp5* (−28 kb) and at an intragenic site (+65 kb) after 1 hr of

OPEN ACCESS
CellPress

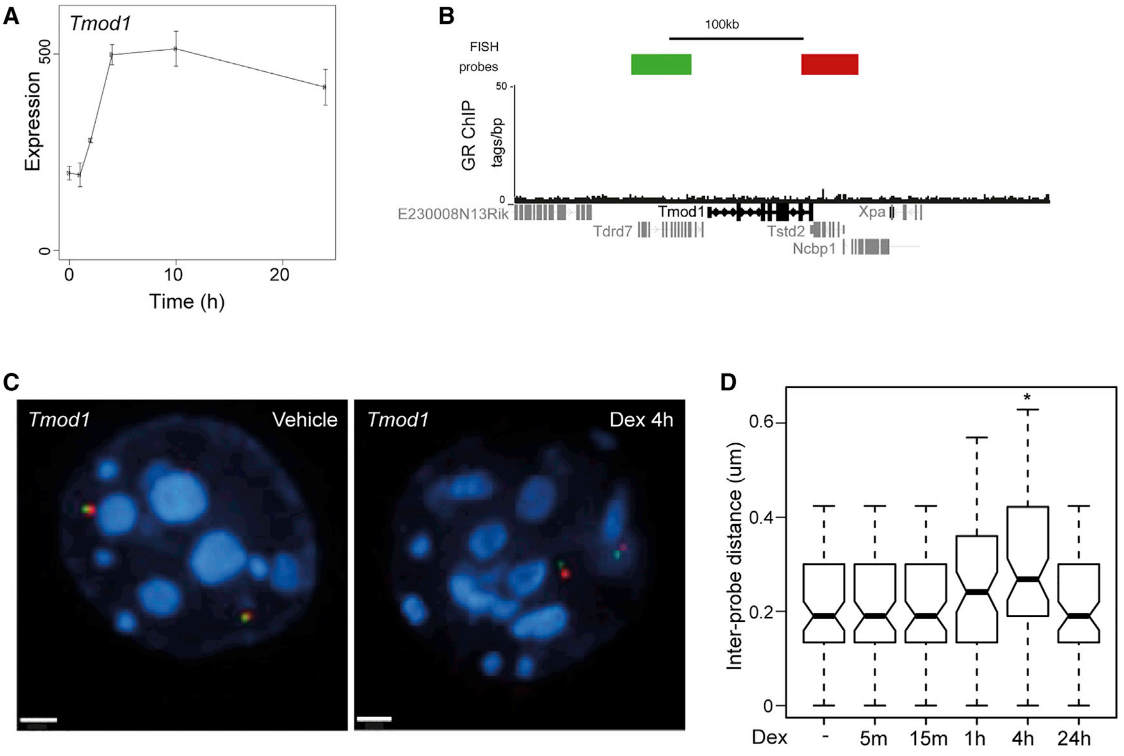

Figure 2. Delayed and Transient Chromatin Decompaction in Response to GC at *Tmod1*

(A) Expression data in response to 100 nM Dex for *Tmod1* at the time points indicated (Jubb et al., 2016). Expression is presented as the raw normalized intensity values from microarrays.

(B) Genome browser image showing GR ChIP-seq data for *Tmod1* locus (Jubb et al., 2016) and the position of probes used for DNA FISH.

(C) 3D DNA FISH images of nuclei from mBMDM 4 hr after treatment with vehicle or 100 nM Dex using the probes indicated in (B). Scale bar represents 1 μm.

(D) Inter-probe distances (μm) measured across *Tmod1* locus at the indicated times following treatment with 100 nM Dex; n = 80 for each condition. Boxplots shown as in Figure 1.

Dex exposure (Figure 1B). As seen for most GC-responsive genes in macrophages from both species (Jubb et al., 2016), no GR binding was detected at the *Fkbp5* promoter. Both GR enhancer locations in the two species correspond to putative enhancers based upon the bidirectional transcription of enhancer-associated enhancer RNA (eRNA) (Andersson et al., 2014). The kinetics of GR binding and loading of chromatin remodeling complexes in cell lines indicate that conformational changes induced by GC may be rapid—within minutes (Johnson et al., 2008; Nagaich et al., 2004; Voss et al., 2011). To examine these kinetics in macrophages, we measured GR binding at the *Fkbp5* locus over a 1-hr time course. There was evidence of GR binding at either enhancer within 5 min of Dex addition. Maximal binding at the −28-kb element occurred by 15 min (Figures 1C and S1B), while binding at +65 kb had slower kinetics, being detectable by 15 min and increasing 1 hr after Dex addition.

GR Binding Is Associated with Rapid and Persistent Chromatin Decompaction at *Fkbp5*

To better understand the relationship between GR binding and long-range chromatin structure, we analyzed chromatin compaction by 3D DNA-FISH (Eskeland et al., 2010; Williamson et al., 2014) using one probe overlapping the −28-kb enhancer and extending to the *Fkbp5* promoter, and another lying just beyond the +65-kb enhancer (Figure 1B). Following Dex treatment of mBMDMs, there was a rapid (<5-min) increase in the average inter-probe distances measured across the *Fkbp5* locus (Figures 1D and 1E). We will refer to this phenomenon as locus decompaction. Figure S2 shows further illustrative images, with and without Dex treatment, from each of the loci tested here. Chromatin decompaction across the *Fkbp5* locus preceded detectable increased stable *Fkbp5* mRNA production (Figure 1A) and was maintained even after 24 hr (Figure 1E). To determine whether chromatin decompaction persists beyond the initial exposure period, Dex was washed out from the culture medium. *Fkbp5* mRNA levels returned to near baseline within 24 hr of washout (Figure 1A). However, chromatin decompaction persisted for 5 days after ligand washout (Figure 1F). Surprisingly, ChIP showed that GR binding at the +65-kb enhancer is transient, decaying completely after 24 hr, regardless of whether Dex was washed out after 1 hr (Figures 1G and S1C). These data suggest that GR binding has a direct and long-lasting effect on large-scale chromatin structure at the *Fkbp5* locus that occurs before the appearance of *Fkbp5* mRNA and persists after *Fkbp5* mRNA is no longer produced, and after GR binding is lost.

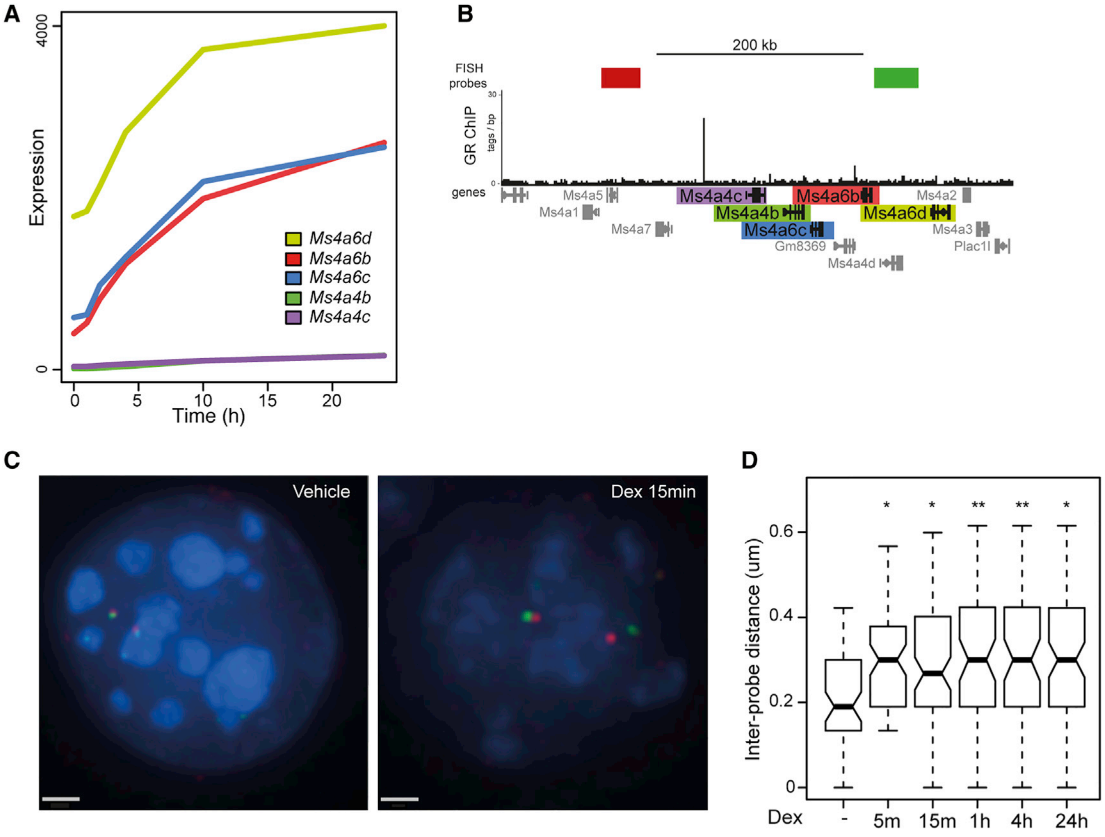

Figure 3. Rapid and Sustained Chromatin Decompaction in Response to GC at a Multi-gene Locus

(A) Expression time course data for the GC-regulated genes in the *Ms4xxx* locus. Expression is presented as the raw normalized intensity values from microarrays.

(B) Genome browser image showing GR ChIP-seq data from mBMDM for *Ms4xxx* locus (Jubb et al., 2016) and the position of probes used for DNA FISH.

(C) 3D DNA FISH images of nuclei from mBMDM 15 min after treatment with vehicle or 100 nM Dex, using the probes indicated in (B). Scale bar represents 1 μm.

(D) Inter-probe distances (μm) measured across the *Ms4xxx* locus at the indicated times following treatment with 100 nM Dex; n = 80 for each condition. Boxplots shown as in Figure 1.

Chromatin Decompaction Is Slow and Transient at a GC-Responsive Locus Where GR Is Not Bound

Most, but not all, GC-inducible genes in mBMDMs have detectable GR bound in their vicinity (±1,000 kb) (Jubb et al., 2016). For responsive genes without detectable GR binding in the general vicinity, transcriptional activation is likely to be a secondary consequence of GC induction of TFs. Eight of the GC-inducible genes in macrophages encode TFs, including four (*Fos*, *Hivep2*, *Klf4*, and *NcoA5*) that were induced within 2 hr and might contribute to downstream target gene induction. The *Tmod1* locus, which shares the same kinetics of gene activation in response to Dex as *Fkbp5* (Figure 2A), did not show evidence of regulated GR binding anywhere within its immediate vicinity (Figure 2B), or indeed anywhere within the wider Mb domain that likely encompasses an entire topologically associated domain (Figure S3) (Dixon et al., 2012). By contrast to the rapid and sustained effect at *Fkbp5*, FISH indicated that chromatin decompaction across the *Tmod1* locus in response to GC occurred more slowly (1–4 hr), paralleled the kinetics of mRNA induction, and was not sustained at 24 hr (Figures 2C and 2D).

Rapid Chromatin Decompaction Occurs at Multiple Loci

Fkbp5 has a specific role in feedback control of the response to GCs, and so could have a unique mode of regulation. We therefore analyzed several other GR-bound loci that differ in whether the response to GC was conserved across species. One of these is a large (720-kb) cluster of genes for the tetraspanin family of transmembrane proteins (referred to here as *Ms4xxx*). A peak of Dex-induced GR binding is located in the center of the *Ms4xxx* cluster, between *Ms4a7* and *Ms4a4c* (Figure 3B). Interestingly, *Ms4a4c* and *Ms4a4b*, close to the GR binding site, are not induced by GC, but the more distant *Ms4a6b*, *Ms4a6c*, and *Ms4a6d* were significantly induced by Dex, albeit more slowly than for *Fkbp5* (Figure 3A). Ms4a family members were also induced in human macrophages, also associated with GR binding to the locus (Jubb et al., 2016). Despite the slower kinetics of gene induction, FISH revealed that, as seen at *Fkbp5*, there was rapid (within 5 min) and sustained chromatin decompaction across the central part of the *Ms4xxx* locus after Dex treatment (Figures 3C and 3D and S2).

A third example of a conserved GR-inducible locus was *Klhl6/B3gnt5/Klhl24* (Figure S4A), where all three transcripts were

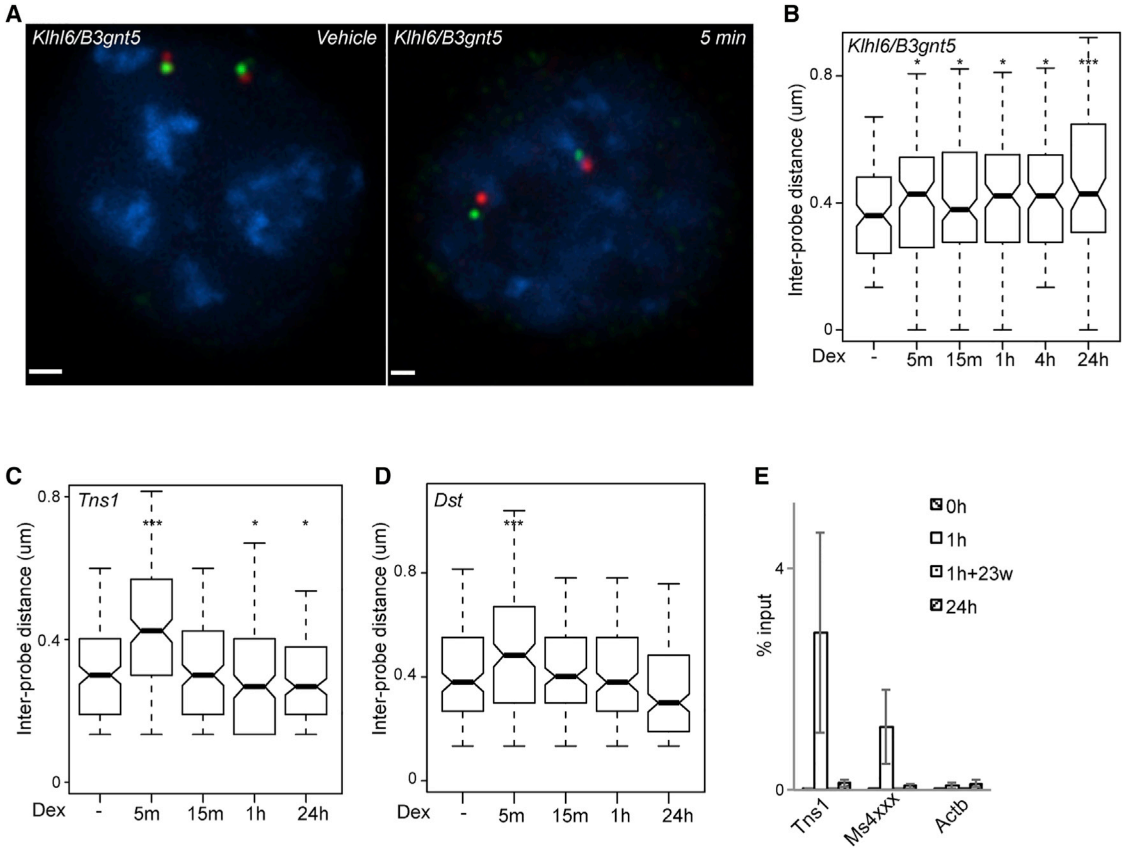

Figure 4. Rapid and Sustained Chromatin Decompaction May Be a Feature of Loci That Are Conserved in Their GC Response

(A) 3D DNA FISH images at the *Klhl6/B3gnt5 locus* of nuclei from mBMDMs 15 min after treatment with vehicle or 100 nM Dex. Probe positions shown in Figure S4A. Scale bar represents 1 μm.

(B–D) Boxplots of the inter-probe distances (μm) measured across the *Klhl6/B3gnt5* (B), *Tns1* (C), and *Dst* (D) loci at the indicated times following treatment with vehicle or 100 nM Dex; n = 120 for each condition. Boxplots as in Figure 1.

(E) GR binding measured by ChIP-qPCR at *Tns1* and *Ms4xxx* loci and *Actb* promoter following stimulation with 100 nM Dex. Data are shown for 1 and 24 hr with (1h+23w) and without (24h) washout of the ligand. Error bars are 2× SEM for three technical replicates (a biological replicate is shown in Figure S5).

induced by Dex. Here again, we observed rapid locus decompaction upon addition of Dex that was sustained for at least 24 hr (Figures 4A and 4B, images from further time points are shown in Figure S2).

We speculated that genes induced by GC only in one species (mouse or human), but not both, might exhibit distinct modes of induction. Accordingly, we examined two loci—*Tns1*, which had a local GR peak, and *Dst*, which did not—induced by Dex in mBMDMs but not in human monocyte-derived macrophages (Figures S4B and S4C). Dex induced rapid chromatin decompaction at these two loci, but by contrast with loci with conserved GC responses between species, this was not sustained beyond the first 5 min (Figures 4C and 4D).

As observed at the *Fkbp5* (+65) enhancer, the sustained chromatin decompaction observed at the *Ms4xxx* locus was not associated with continuous GR binding. GR binding was undetectable at both the *Ms4xxx* and *Tns1* loci after 24 hr, regardless of whether or not the agonist was washed out after 1 hr (Figures 4E and S5).

Rapid Chromatin Decompaction at GR-Bound Loci Can Occur without Transcription

Chromatin at actively transcribed regions is generally less compact than at silenced regions (Chambeyron and Bickmore 2004; Naughton et al., 2013). Therefore, the decompaction we observed in response to Dex could be a consequence of, rather than causally linked to, transcriptional activation induced by GR. The rapidity of the response (within 5 min) and the persistence long after loss of GR binding (Figure 4E) and target gene inactivation, argue against the simple proposition that chromatin decompaction is associated with the act of transcription. However, to test this directly, we used α-amanitin to inhibit transcription by RNA Polymerase II. At *Tmod1*, where the induction by Dex is presumed to be indirect given the lack of GR binding (Figures 2B and S3), pre-treatment of mBMDM with α-amanitin for 4 hr ablated the Dex-dependent changes in chromatin compaction (Figures 5A and 5B). Unexpectedly, α-amanitin also prevented detectable chromatin changes at *Klhl6/B3gnt5* (Figure 5C). This suggests either that this response is associated with transcription directly, or that it depends upon a labile regulator that decays, or is not

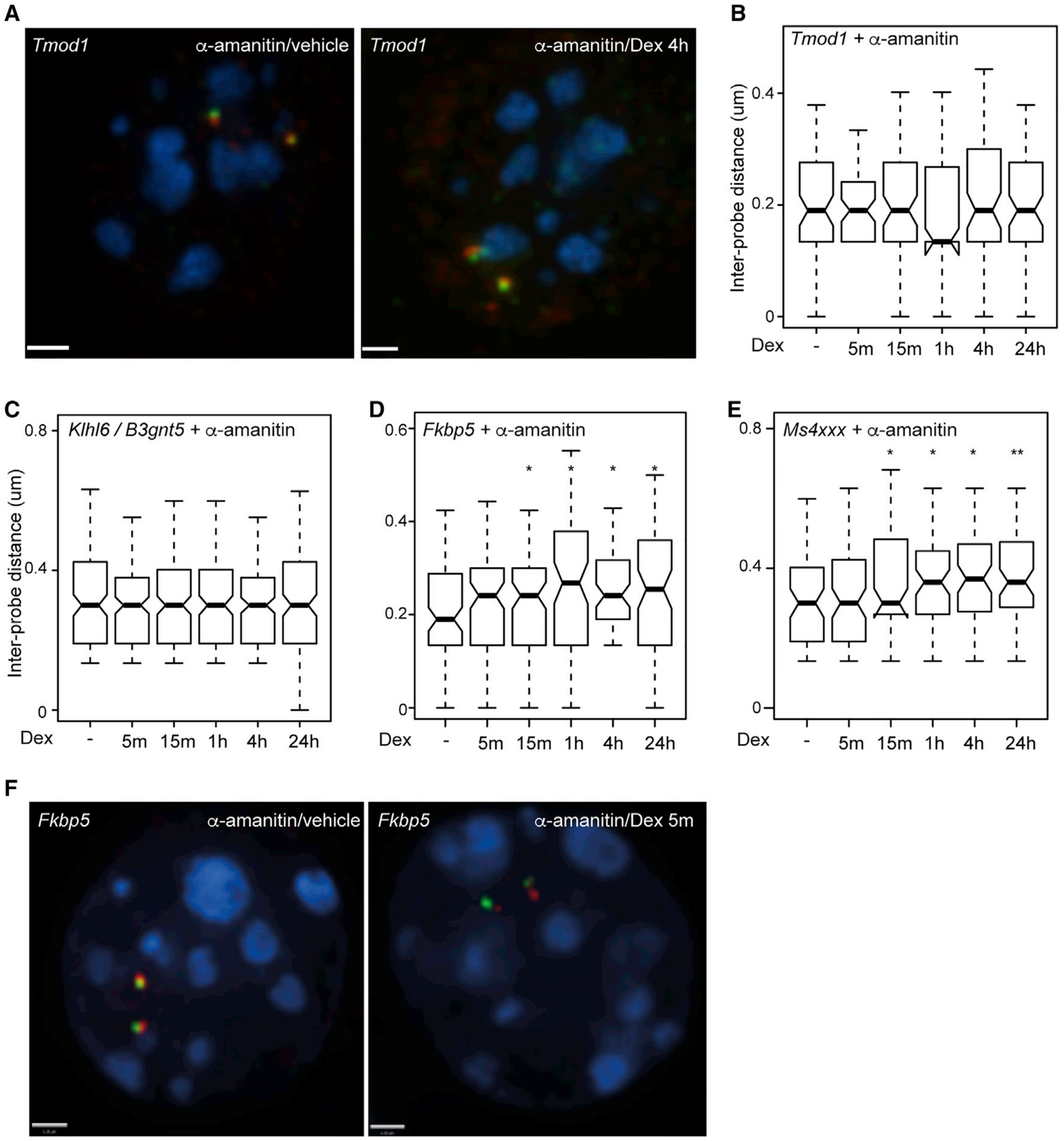

Figure 5. α-Amanitin Does Not Block Rapid Chromatin Unfolding at GR-Bound Loci

(A) 3D DNA FISH images using probes across the *Tmod1* locus (Figure 2A) in nuclei from mBMDM that have been pretreated with 2.5 μg/mL α-amanitin for 4 hr, and then treated with 100 nM Dex for the indicated time. Scale bar represents 1 μm.

(B) Inter-probe distances measured across *Tmod1* locus after pretreatment with α-amanitin for 4 hr and then treatment with 100 nM Dex for the indicated times. n = 120 for each dataset. *p < 0.05, Wilcoxon rank sum.

(C–E) Analogous to (B) but measured across the (C) *Klhl6/B3gnt5*, (D) *Fkbp5*, and (E) *Ms4xxx* loci.

(F) 3D DNA FISH images using probes across the *Fkbp5* locus (Figure 1B) showing nuclei from mBMDMs that have been pretreated with 2.5 μg/mL α-amanitin for 4 hr, and then treated with 100 nM Dex for the indicated time. Scale bar represents 1 μm.

produced, in the presence of the inhibitor (Stacey et al., 1994). Although the GR-bound enhancer at the *Klhl6/B3gnt5* locus has conserved GR and PU.1 consensus motifs, its GR-ChIP-seq peak is weaker than those at the *Fkbp5* and *Ms4xxx* loci. By contrast, Dex still induced significant chromatin decompaction at the *Fkbp5* (Figures 5D and 5F) and the *Ms4xxx* loci (Figure 5E) in the presence of α-amanitin, albeit with slightly delayed kinetics.

DISCUSSION

Single-cell imaging indicates that the binding of the GR, and associated coactivators that recruit chromatin remodeling complexes (GRIP1, BRG1), is dynamic and rapidly reversible (Paakinaho et al., 2017). In the present study, we have shown that GC produces very rapid changes to higher-order

Table 1. Primers

Locus	Sequence
Fkbp5 −28 kb, forward	GAACACAGTGTCCCCCAGAG
Fkbp5 −28 kb, reverse	CAGGAGAGGAGGAGAGGGTC
Fkbp5 promoter, forward	TTTGCATCTCCGCCTCTTCA
Fkbp5 promoter, reverse	TCCTCCATCCCTCTTCTCCG
Fkbp5 +65 kb, forward	GCCAAGTTCAGCTGTGCAAT
Fkbp5 +65 kb, reverse	TGCCAGCCACATTCAGAACA
Tns1, forward	GCAGTTTGGAGCCAAAAAGACC
Tns1, reverse	TGGGTCTGAGCAATTCCAGTTC
Ms4xxx, forward	TGTTAATGGTGGCGTGAGAGTG
Ms4xxx, reverse	ATAAGACGTGGTACTGCCTGAG
Actb promoter, forward	CTAGCCACGAGAGAGCGAAG
Actb promoter, reverse	CGCGAGCACAGCTTCTTT

chromatin structure in macrophages at genomic sites where GR binds directly to DNA. Moreover, these changes persist for prolonged periods beyond the exposure to GC and do not depend upon the continuous presence of bound GR. It has been suggested that GR occupancy patterns in different cell types are predetermined by the distinctive baseline nucleosome accessibility patterns, a significant minority of which are then altered after GC treatment (John et al., 2011). Local transient GR binding can promote subsequent binding of a variant GR to the same motif, a phenomenon referred to as "assisted loading" (Voss et al., 2011).

Sustained chromatin reorganization in response to GR is not universal among target loci. The three genes tested that were induced only in mice, *Tns1*, *Tmod1*, and *Dst*, showed decompaction in response to Dex, but this was not sustained (Figures 4C and 4D), even though one of these (*Tns1*) has a GR-bound enhancer. The observation that most, but not all, inducible DNase I hypersensitive sites (DHSs) revert to the pre-stimulation state following GR stimulation (Stavreva et al., 2015) suggests that these genes are in the majority.

The rapid chromatin decompaction we report at multiple GR-responsive loci is distinct from previous reports. Transient chromatin reorganization of the MMTV array detected following GC exposure could be prevented by blocking transcription (Müller et al., 2001). However, lack of effect of α-amanitin at one-half of the loci we studied (Figure 5) suggests that transcription, per se, is not absolutely required for GR-induced decompaction.

At first sight, GR-induced chromatin decompaction at the *Fkbp5* locus conflicts with evidence of the formation of a compact loop domain in GC-responsive loci inferred in other systems (Paakinaho et al., 2010; Klengel et al., 2013). However, chromosome conformation capture and FISH assays can give apparently discordant views of spatial genome organization (Fraser et al., 2015; Williamson et al., 2014) and chromatin decompaction could still be permissive for transient, rather than stable, interactions between elements that can be captured by 3C methods. We cannot exclude that the decompaction we observe represents GC-induced dissolution of preformed chromatin loops. The action of the GR that we report here is reminiscent of large-scale chromatin decompaction induced by other nuclear hormone receptors: the ecdysone receptor, which induces visible chromatin decompaction (puffing) on *Drosophila* polytene chromosomes (Tulin and Spradling, 2003; Sawatsubashi et al., 2004), and the estrogen receptor (Nye et al., 2002; Rafique et al., 2015). The molecular details of these decompact chromatin structures are yet to be elucidated.

The loss of bound GR from target loci, despite the continued presence of the agonist, is reminiscent of the state of tolerance elicited in macrophages by lipopolysaccharide (Seeley and Ghosh 2017). Most explanations for GC resistance in human patients are based upon the regulation of cellular responsiveness by extrinsic signals, such as inflammatory cytokines and bacterial products (Bekhbat et al., 2017; Dendoncker and Libert 2017; Silverman et al., 2013; Silverman and Sternberg 2012). Consistent with that view, the response of the mouse BMDMs studied herein, and by others (Oh et al., 2017), is acutely regulated by CSF-1 (Hume and Gordon 1984). Persistent changes in chromatin structure, and a failure to rapidly return to the chromatin ground state that existed before GC exposure, suggest an additional mechanism that may contribute. Chromatin reorganization at key target loci in response to GR binding could alter the likelihood of those loci being regulated by a future stimulus. However, the effects of GC are often context specific (Klengel et al., 2013) and primary macrophages in culture evolve over time. It remains to be seen whether sustained chromatin decompaction can be observed in monocyte/macrophages derived from GC-treated patients and whether this is associated with either therapeutic efficacy, or the development of GC resistance.

EXPERIMENTAL PROCEDURES

Ethics

Animals were cared for and managed within the Roslin Institute's Biological Research Facility following Institute guidelines. The Roslin Institute is committed to the highest standards of animal welfare and the University of Edinburgh is a signatory of the Concordat on Openness on Animals in Research in the UK. No interventions were performed on live animals for this research.

Cell Culture

The 8- to 10-week male wild-type C57BL/6 mice were culled by cervical dislocation. Bone marrow was flushed from hindlimbs and then cultured in RPMI supplemented with penicillin/streptomycin, Glutamax (Invitrogen), and 10% fetal calf serum for 7 days in the presence of 10^4 U/mL rhCSF-1. The resulting mBMDMs were replated onto Superfrost microscope slides (Thermo) at 5×10^5 cells/mL and treated as indicated with 100 nM Dex (Sigma) or ethanol vehicle. Where described, cells were pre-treated with 2.5 μg/mL α-amanitin (Sigma) for 4 hr. Dex was washed out by removal of ligand-containing medium, washing gently once with fresh medium and then returning to culture in further fresh medium.

Chromatin Immunoprecipitation

Antibodies used for chromatin immunoprecipitation of mouse GR were BuGR2 (1 μg/10^6 cells; Thermo Fisher/Pierce) and rabbit IgG sc-2025 (Santa Cruz).

To prepare antibody-bound beads, 20 μL of Protein A Dynabeads (Invitrogen) per immunoprecipitation (IP) were washed once, and then diluted to 200 μL in block solution (1× PBS, 0.5% BSA, +2 μL of 0.1 M PMSF). Antibody was added and rotated for 3 hr at 4°C.

Table 2. Fosmid Clones

Locus	Whitehead Fosmid Name	Map Position (Chromosome: Sequence Range)
Fkbp5	WI1-1951C9	chr17:28621896–28660104
Fkbp5	WI1-980F19	chr17:28504480–28545426
Tmod1	WI1-2441L4	chr4:46121795–46162130
Tmod1	WI1-552C3	chr4:45995494–46038724
Ms4xxx	WI1-1714F1	chr19:11344410–11380699
Ms4xxx	WI1-794B24	chr19:11607184–11647412
Klhl6	WI1-593E13	chr16:19778756–19815279
Klhl6	WI1-1540E20	chr16:19986135–20022250
Dst	WI1-2074K18	chr1:34007075–34047561
Dst	WI1-1520P06	chr1:34311167–34352486
Tns1	WI1-2221B20	chr1:74136897–74174845
Tns1	WI1-501O4	chr1:73867180–73908234

Cells were washed gently once with PBS, cross-linked in tissue culture plates with 1% formaldehyde/RPMI at room temperature for 10 min, and then quenched with 0.125 M glycine. Cells were detached by scraping in PBS, and then spun down (400 × *g*, 5 min, 4°C), resuspended, and counted. 10^6 cells per IP were lysed for 15 min on ice in 1% SDS, 10 mM EDTA, 50 mM Tris-HCl, pH 8.1, supplemented with protease inhibitors (Calbiochem), 1 mM DTT, and 0.2 mM PMSF (Sigma). The solution was diluted in IP dilution buffer (0.1% Triton X-100, 2 mM EDTA, 150 mM NaCl, 20 mM Tris-HCl, pH 8.1) and sonicated using a Soniprep 150 to produce 300- to 500-bp average fragment sizes. Chromatin was spun for 10 min at 10,000 × *g* (4°C), and then supplemented with 20% Triton X-100 to 1%, and BSA (Sigma) to 50 μg/mL. Input aliquots were removed and stored at −20°C. Chromatin was then added to the antibody-bound Protein A Dynabeads (Life Technologies) and rotated overnight at 4°C. After binding, beads were washed 3 × 10 min each in the following: (1) 1% IP dilution buffer; (2) 1% Triton X-100/0.1% Na-deoxycholate/0.1% SDS, 50 mM HEPES, pH 7.9, 500 mM NaCl, 1 mM EDTA; and (3) 0.5% Na-deoxycholate/0.5% NP-40, 20 mM Tris-HCl, pH 8, 1 mM EDTA, and 250 mM LiCl. Chromatin was extracted at 37°C for 15 min on a vibrating platform in 100 μL of extraction buffer (0.1 M $NaHCO_3$, 1% SDS). To reverse cross-links, samples were supplemented to 300 mM with NaCl, treated with RNaseA (20 mg) (Roche), and then incubated for ~8 hr at 65°C. Proteinase K (40 μg) (Genaxxon) was added, and samples were incubated at 55°C for 1 hr. DNA was purified using the QIAquick PCR purification kit (QIAGEN). Real-time qPCR analysis to determine percent input bound at known GR target loci was carried out on a LightCycler 480 System using SYBR Green Master Mix (Roche).

Primers

Primers are listed in Table 1.

3D DNA FISH

Paraformaldehyde (pFA)-fixed cells were permeabilized in 0.5% Triton X-100, washed in PBS, and stored at −80°C. 3D-FISH was carried out as previously described (Eskeland et al., 2010). Slides were imaged and analyzed as described previously (Williamson et al., 2012). The statistical significance of differences in n (values in figure legends) measured inter-probe distances was assessed using the nonparametric Wilcoxon rank sum test in R. Fosmid clones were from BACPAC Resource Center (Oakland, CA) and are listed in Table 2.

DATA AND SOFTWARE AVAILABILITY

The accession number for the microarray and ChIP sequencing data reported in this study (from Jubb et al., 2016) is GEO: GSE61881.

SUPPLEMENTAL INFORMATION

Supplemental Information includes five figures and can be found with this article online at https://doi.org/10.1016/j.celrep.2017.11.053.

ACKNOWLEDGMENTS

A.W.J. was supported by a Wellcome Trust Clinical PhD Fellowship (097481/Z/11/Z). W.A.B. and S.B. are supported by University Unit Programme Grant U127527202 from the UK Medical Research Council. The Roslin Institute is supported by Institute Strategic Programme Grants from BBSRC. We are grateful for the assistance of Clare Pridans (Roslin Institute) in the preparation of macrophages for some of the experiments.

AUTHOR CONTRIBUTIONS

A.W.J. designed the study, performed the experiments, and wrote the paper. S.B. performed experiments. D.A.H. and W.A.B. designed the study and wrote the paper.

Received: December 15, 2015
Revised: October 1, 2017
Accepted: November 15, 2017
Published: December 12, 2017

REFERENCES

Andersson, R., Gebhard, C., Miguel-Escalada, I., Hoof, I., Bornholdt, J., Boyd, M., Chen, Y., Zhao, X., Schmidl, C., Suzuki, T., et al. (2014). An atlas of active enhancers across human cell types and tissues. Nature *507*, 455–461.

Annane, D., Sébille, V., Troché, G., Raphaël, J.C., Gajdos, P., and Bellissant, E. (2000). A 3-level prognostic classification in septic shock based on cortisol levels and cortisol response to corticotropin. JAMA *283*, 1038–1045.

Bekhbat, M., Rowson, S.A., and Neigh, G.N. (2017). Checks and balances: the glucocorticoid receptor and NFκB in good times and bad. Front. Neuroendocrinol. *46*, 15–31.

Belikov, S., Astrand, C., and Wrange, O. (2009). FoxA1 binding directs chromatin structure and the functional response of a glucocorticoid receptor-regulated promoter. Mol. Cell. Biol. *29*, 5413–5425.

Biddie, S.C., John, S., Sabo, P.J., Thurman, R.E., Johnson, T.A., Schiltz, R.L., Miranda, T.B., Sung, M.H., Trump, S., Lightman, S.L., et al. (2011). Transcription factor AP1 potentiates chromatin accessibility and glucocorticoid receptor binding. Mol. Cell *43*, 145–155.

Boonen, E., Vervenne, H., Meersseman, P., Andrew, R., Mortier, L., Declercq, P.E., Vanwijngaerden, Y.M., Spriet, I., Wouters, P.J., Vander Perre, S., et al. (2013). Reduced cortisol metabolism during critical illness. N. Engl. J. Med. *368*, 1477–1488.

Burd, C.J., and Archer, T.K. (2013). Chromatin architecture defines the glucocorticoid response. Mol. Cell. Endocrinol. *380*, 25–31.

Chambeyron, S., and Bickmore, W.A. (2004). Chromatin decondensation and nuclear reorganization of the HoxB locus upon induction of transcription. Genes Dev. *18*, 1119–1130.

Dendoncker, K., and Libert, C. (2017). Glucocorticoid resistance as a major drive in sepsis pathology. Cytokine Growth Factor Rev. *35*, 85–96.

Denny, W.B., Valentine, D.L., Reynolds, P.D., Smith, D.F., and Scammell, J.G. (2000). Squirrel monkey immunophilin FKBP51 is a potent inhibitor of glucocorticoid receptor binding. Endocrinology *141*, 4107–4113.

Dixon, J.R., Selvaraj, S., Yue, F., Kim, A., Li, Y., Shen, Y., Hu, M., Liu, J.S., and Ren, B. (2012). Topological domains in mammalian genomes identified by analysis of chromatin interactions. Nature *485*, 376–380.

Eskeland, R., Leeb, M., Grimes, G.R., Kress, C., Boyle, S., Sproul, D., Gilbert, N., Fan, Y., Skoultchi, A.I., Wutz, A., and Bickmore, W.A. (2010). Ring1B

compacts chromatin structure and represses gene expression independent of histone ubiquitination. Mol. Cell *38*, 452–464.

Forrest, A.R., Kawaji, H., Rehli, M., Baillie, J.K., de Hoon, M.J., Haberle, V., Lassmann, T., Kulakovskiy, I.V., Lizio, M., Itoh, M., et al.; FANTOM Consortium and the RIKEN PMI and CLST (DGT) (2014). A promoter-level mammalian expression atlas. Nature *507*, 462–470.

Fraser, J., Williamson, I., Bickmore, W.A., and Dostie, J. (2015). An overview of genome organization and how we got there: from FISH to Hi-C. Microbiol. Mol. Biol. Rev. *79*, 347–372.

Grøntved, L., John, S., Baek, S., Liu, Y., Buckley, J.R., Vinson, C., Aguilera, G., and Hager, G.L. (2013). C/EBP maintains chromatin accessibility in liver and facilitates glucocorticoid receptor recruitment to steroid response elements. EMBO J. *32*, 1568–1583.

Hakim, O., Sung, M.H., Voss, T.C., Splinter, E., John, S., Sabo, P.J., Thurman, R.E., Stamatoyannopoulos, J.A., de Laat, W., and Hager, G.L. (2011). Diverse gene reprogramming events occur in the same spatial clusters of distal regulatory elements. Genome Res. *21*, 697–706.

Hume, D.A., and Gordon, S. (1984). The correlation between plasminogen activator activity and thymidine incorporation in mouse bone marrow-derived macrophages. Opposing actions of colony-stimulating factor, phorbol myristate acetate, dexamethasone and prostaglandin E. Exp. Cell Res. *150*, 347–355.

Jääskeläinen, T., Makkonen, H., and Palvimo, J.J. (2011). Steroid up-regulation of FKBP51 and its role in hormone signaling. Curr. Opin. Pharmacol. *11*, 326–331.

John, S., Sabo, P.J., Johnson, T.A., Sung, M.H., Biddie, S.C., Lightman, S.L., Voss, T.C., Davis, S.R., Meltzer, P.S., Stamatoyannopoulos, J.A., and Hager, G.L. (2008). Interaction of the glucocorticoid receptor with the chromatin landscape. Mol. Cell *29*, 611–624.

John, S., Sabo, P.J., Thurman, R.E., Sung, M.H., Biddie, S.C., Johnson, T.A., Hager, G.L., and Stamatoyannopoulos, J.A. (2011). Chromatin accessibility pre-determines glucocorticoid receptor binding patterns. Nat. Genet. *43*, 264–268.

Johnson, T.A., Elbi, C., Parekh, B.S., Hager, G.L., and John, S. (2008). Chromatin remodeling complexes interact dynamically with a glucocorticoid receptor-regulated promoter. Mol. Biol. Cell *19*, 3308–3322.

Jubb, A.W., Young, R.S., Hume, D.A., and Bickmore, W.A. (2016). Enhancer turnover is associated with a divergent transcriptional response to glucocorticoid in mouse and human macrophages. J. Immunol. *196*, 813–822.

Klengel, T., Mehta, D., Anacker, C., Rex-Haffner, M., Pruessner, J.C., Pariante, C.M., Pace, T.W., Mercer, K.B., Mayberg, H.S., Bradley, B., et al. (2013). Allele-specific FKBP5 DNA demethylation mediates gene-childhood trauma interactions. Nat. Neurosci. *16*, 33–41.

Lattin, J.E., Schroder, K., Su, A.I., Walker, J.R., Zhang, J., Wiltshire, T., Saijo, K., Glass, C.K., Hume, D.A., Kellie, S., and Sweet, M.J. (2008). Expression analysis of G protein-coupled receptors in mouse macrophages. Immunome Res. *4*, 5.

Lim, H.W., Uhlenhaut, N.H., Rauch, A., Weiner, J., Hübner, S., Hübner, N., Won, K.J., Lazar, M.A., Tuckermann, J., and Steger, D.J. (2015). Genomic redistribution of GR monomers and dimers mediates transcriptional response to exogenous glucocorticoid in vivo. Genome Res. *25*, 836–844.

Müller, W.G., Walker, D., Hager, G.L., and McNally, J.G. (2001). Large-scale chromatin decondensation and recondensation regulated by transcription from a natural promoter. J. Cell Biol. *154*, 33–48.

Nagaich, A.K., Walker, D.A., Wolford, R., and Hager, G.L. (2004). Rapid periodic binding and displacement of the glucocorticoid receptor during chromatin remodeling. Mol. Cell *14*, 163–174.

Naughton, C., Avlonitis, N., Corless, S., Prendergast, J.G., Mati, I.K., Eijk, P.P., Cockroft, S.L., Bradley, M., Ylstra, B., and Gilbert, N. (2013). Transcription forms and remodels supercoiling domains unfolding large-scale chromatin structures. Nat. Struct. Mol. Biol. *20*, 387–395.

Nye, A.C., Rajendran, R.R., Stenoien, D.L., Mancini, M.A., Katzenellenbogen, B.S., and Belmont, A.S. (2002). Alteration of large-scale chromatin structure by estrogen receptor. Mol. Cell. Biol. *22*, 3437–3449.

Oh, K., Patel, H., Gottschalk, R.A., Lee, W.S., Baek, S., Fraser, I.D.C., Hager, G.L., and Sung, M.H. (2017). Anti-inflammatory chromatinscape suggests alternative mechanisms of glucocorticoid receptor action. Immunity *47*, 298–309.e5.

Ostuni, R., Piccolo, V., Barozzi, I., Polletti, S., Termanini, A., Bonifacio, S., Curina, A., Prosperini, E., Ghisletti, S., and Natoli, G. (2013). Latent enhancers activated by stimulation in differentiated cells. Cell *152*, 157–171.

Paakinaho, V., Makkonen, H., Jääskeläinen, T., and Palvimo, J.J. (2010). Glucocorticoid receptor activates poised FKBP51 locus through long-distance interactions. Mol. Endocrinol. *24*, 511–525.

Paakinaho, V., Presman, D.M., Ball, D.A., Johnson, T.A., Schiltz, R.L., Levitt, P., Mazza, D., Morisaki, T., Karpova, T.S., and Hager, G.L. (2017). Single-molecule analysis of steroid receptor and cofactor action in living cells. Nat. Commun. *8*, 15896.

Rafique, S., Thomas, J.S., Sproul, D., and Bickmore, W.A. (2015). Estrogen-induced chromatin decondensation and nuclear re-organization linked to regional epigenetic regulation in breast cancer. Genome Biol. *16*, 145.

Reddy, T.E., Pauli, F., Sprouse, R.O., Neff, N.F., Newberry, K.M., Garabedian, M.J., and Myers, R.M. (2009). Genomic determination of the glucocorticoid response reveals unexpected mechanisms of gene regulation. Genome Res. *19*, 2163–2171.

Rodriguez, J.M., Monsalves-Alvarez, M., Henriquez, S., Llanos, M.N., and Troncoso, R. (2016). Glucocorticoid resistance in chronic diseases. Steroids *115*, 182–192.

Sawatsubashi, S., Maki, A., Ito, S., Shirode, Y., Suzuki, E., Zhao, Y., Yamagata, K., Kouzmenko, A., Takeyama, K., and Kato, S. (2004). Ecdysone receptor-dependent gene regulation mediates histone poly(ADP-ribosyl)ation. Biochemi. Biophys. Res. Commun *320*, 268–272.

Seeley, J.J., and Ghosh, S. (2017). Molecular mechanisms of innate memory and tolerance to LPS. J. Leukoc. Biol. *101*, 107–119.

Silverman, M.N., and Sternberg, E.M. (2012). Glucocorticoid regulation of inflammation and its functional correlates: from HPA axis to glucocorticoid receptor dysfunction. Ann. N Y Acad. Sci. *1261*, 55–63.

Silverman, M.N., Mukhopadhyay, P., Belyavskaya, E., Tonelli, L.H., Revenis, B.D., Doran, J.H., Ballard, B.E., Tam, J., Pacher, P., and Sternberg, E.M. (2013). Glucocorticoid receptor dimerization is required for proper recovery of LPS-induced inflammation, sickness behavior and metabolism in mice. Mol. Psychiatry *18*, 1006–1017.

So, A.Y.-L., Chaivorapol, C., Bolton, E.C., Li, H., and Yamamoto, K.R. (2007). Determinants of cell- and gene-specific transcriptional regulation by the glucocorticoid receptor. PLoS Genet. *3*, e94.

Stacey, K.J., Nagamine, Y., and Hume, D.A. (1994). RNA synthesis inhibition stabilises urokinase mRNA in macrophages. FEBS Lett. *356*, 311–313.

Starick, S.R., Ibn-Salem, J., Jurk, M., Hernandez, C., Love, M.I., Chung, H.R., Vingron, M., Thomas-Chollier, M., and Meijsing, S.H. (2015). ChIP-exo signal associated with DNA-binding motifs provides insight into the genomic binding of the glucocorticoid receptor and cooperating transcription factors. Genome Res. *25*, 825–835.

Stavreva, D.A., Coulon, A., Baek, S., Sung, M.H., John, S., Stixova, L., Tesikova, M., Hakim, O., Miranda, T., Hawkins, M., et al. (2015). Dynamics of chromatin accessibility and long-range interactions in response to glucocorticoid pulsing. Genome Res. *25*, 845–857.

Tulin, A., and Spradling, A. (2003). Chromatin loosening by poly(ADP)-ribose polymerase (PARP) at Drosophila puff loci. Science *299*, 560–562.

Uhlenhaut, N.H., Barish, G.D., Yu, R.T., Downes, M., Karunasiri, M., Liddle, C., Schwalie, P., Hübner, N., and Evans, R.M. (2013). Insights into negative regulation by the glucocorticoid receptor from genome-wide profiling of inflammatory cistromes. Mol. Cell *49*, 158–171.

Vandevyver, S., Dejager, L., and Libert, C. (2012). On the trail of the glucocorticoid receptor: into the nucleus and back. Traffic *13*, 364–374.

Voss, T.C., Schiltz, R.L., Sung, M.H., Yen, P.M., Stamatoyannopoulos, J.A., Biddie, S.C., Johnson, T.A., Miranda, T.B., John, S., and Hager, G.L. (2011). Dynamic exchange at regulatory elements during chromatin remodeling underlies assisted loading mechanism. Cell *146*, 544–554.

Williamson, I., Eskeland, R., Lettice, L.A., Hill, A.E., Boyle, S., Grimes, G.R., Hill, R.E., and Bickmore, W.A. (2012). Anterior-posterior differences in HoxD chromatin topology in limb development. Development *139*, 3157–3167.

Williamson, I., Berlivet, S., Eskeland, R., Boyle, S., Illingworth, R.S., Paquette, D., Dostie, J., and Bickmore, W.A. (2014). Spatial genome organization: contrasting views from chromosome conformation capture and fluorescence in situ hybridization. Genes Dev. *28*, 2778–2791.

Zannas, A.S., Wiechmann, T., Gassen, N.C., and Binder, E.B. (2016). Gene-stress-epigenetic regulation of FKBP5: clinical and translational implications. Neuropsychopharmacology *41*, 261–274.

Cell Reports, Volume *21*

Supplemental Information

Glucocorticoid Receptor Binding Induces Rapid and Prolonged Large-Scale Chromatin Decompaction at Multiple Target Loci

Alasdair W. Jubb, Shelagh Boyle, David A. Hume, and Wendy A. Bickmore

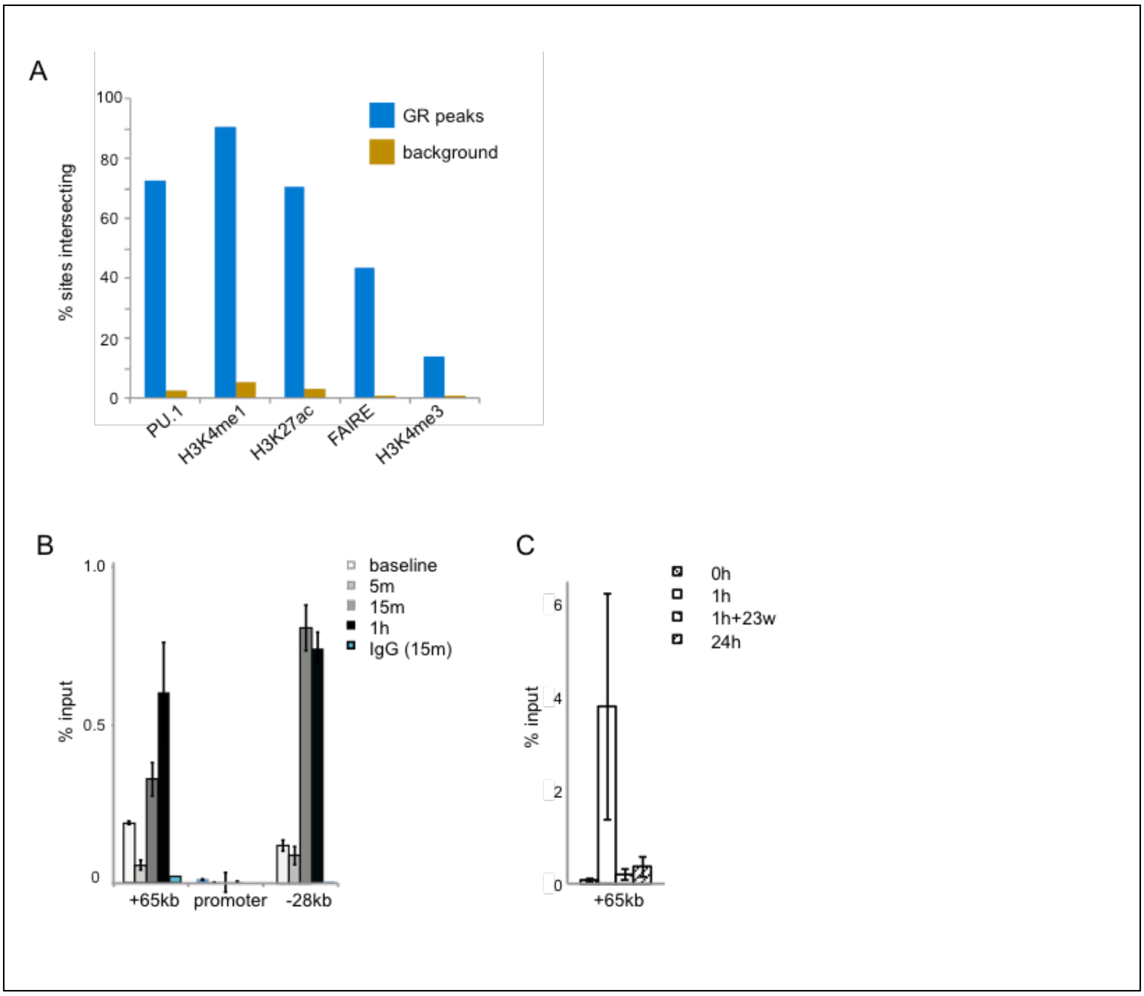

Figure S1. GR binding precedes detectable rises in *Fkbp5* mRNA and is lost by 24h. Related to Fig 1. (A) Percent of GR binding sites in mBMDM treated with Dex that overlap with the given features in un-treated mBMDMs. Comparison is between our previous GR data (Jubb et al., 2016) and published data for PU.1 binding, H3K4me1, H3K27ac, FAIRE-seq and H3K4me3 (Ostuni et al., 2013). Blue; data for GR peaks. Orange; data for a GC matched genome permuted background set of peaks. (B) Glucocorticoid receptor binding in Dex treated mBMDM measured by ChIP-qPCR for the downstream enhancer (+65kb), promoter and upstream enhancer (-28kb) of *Fkbp5*. Data is shown for a 4 point time series (baseline, 5min, 15min,1h) of treatment with 100nM dexamethasone. Normal IgG is also shown. Error bars are 2 x standard error of the mean (SEM) for 3 technical replicates. This data is a biological replicate of the data shown in Figure 1C. (C) Glucocorticoid receptor binding measured by ChIP-qPCR for the downstream enhancer (Fkbp5+65kb) following stimulation with 100nM dexamethasone at 1 hour and 24h with (1h+23w) and without (24h) washout of the ligand. Error bars are 2 x standard error of the mean (SEM) for 3 technical replicates. This is a biological replicate of the data in Figure 1G.

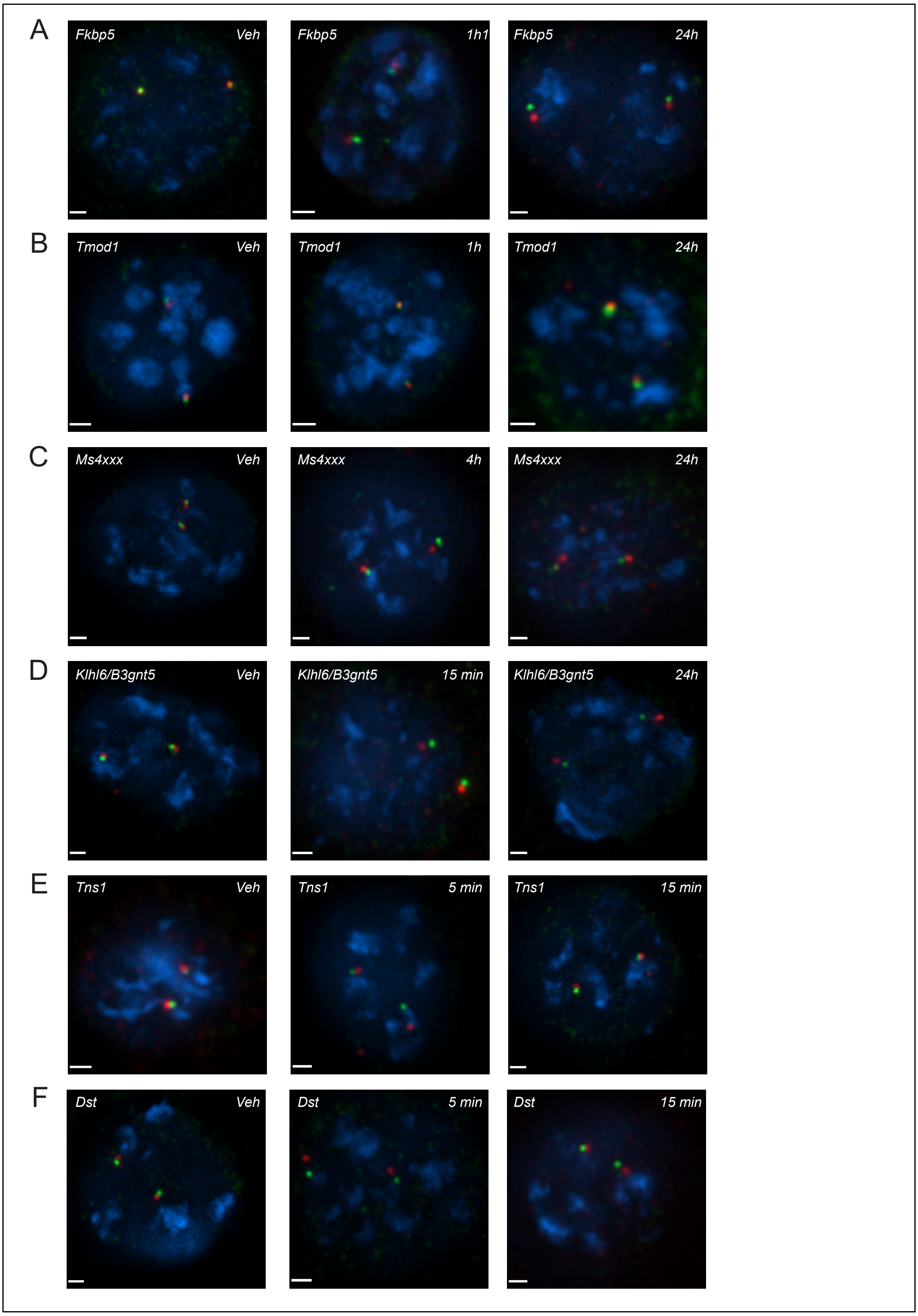

Figure S2. Illustrative DNA FISH images. Related to Figures 1, 2, 3 and 4. (A-F) Images of DNA FISH from each of the studied loci at the time points indicated. These images are presented for illustration purposes and represent merged 3D image stacks. Data collection was performed in 3D as described previously (Eskeland et al., 2010).

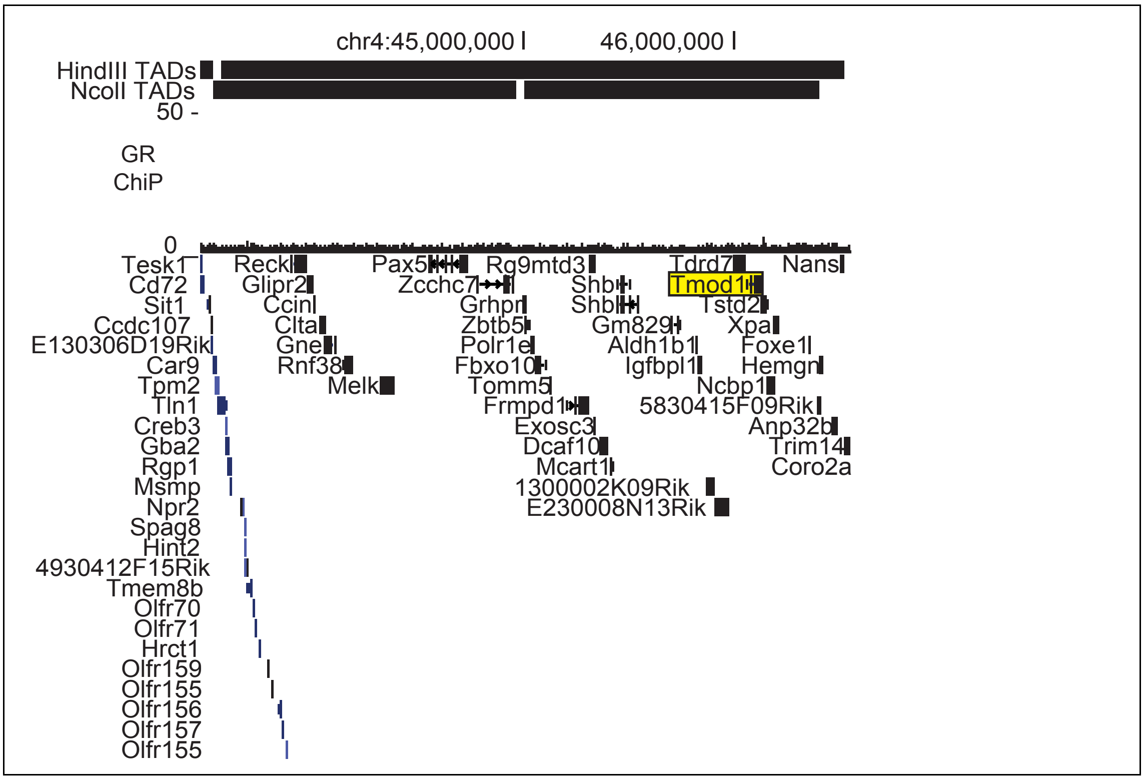

Figure S3. *Tmod1* does not have GR bound in the same TAD. Related to Figure 2. Very wide view from the UCSC genome browser of the *Tmod1* locus that shows no GR bound within either of the two sets of Topology Associated Domains (TADs) reported in (Dixon et al., 2012) using HindIII and NcoII restriction enzymes, TADs shown in black above locus.

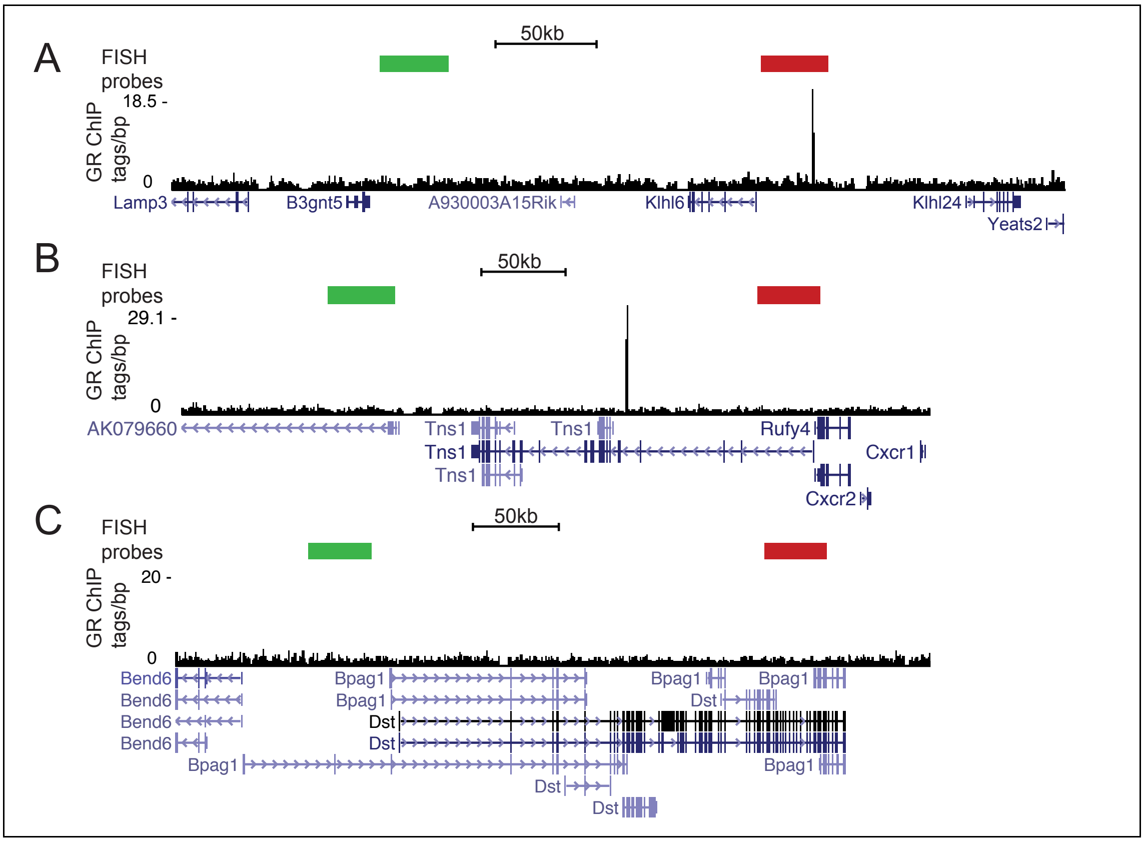

Figure S4. Locations of additional fosmid probes used for 3D DNA FISH. **Related to Figures 1, 2, 3 and 4**. (A) UCSC Genome browser image showing *Klhl6 / B3gnt5 / Klhl24* gene locus with ChIP-seq data (tags/bp) for GR binding from (Jubb et al. 2016). Red and green blocks show the positions of fosmid probes used for FISH analysis (mm9). (B & C) analogous images to (A) for *Tns1* and *Dst.*

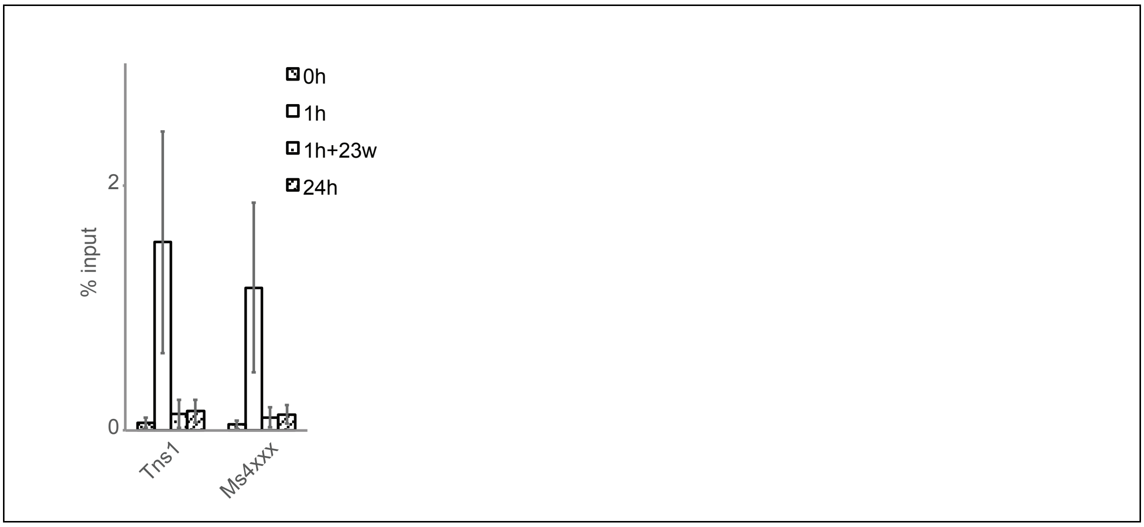

Figure S5. Glucocorticoid receptor binding is not present at two further target loci 24h after treatment. Related to Figure 4. Glucocorticoid receptor binding in Dex treated mBMDM measured by ChIP-qPCR at *Tns1* and *Ms4xxx* loci following stimulation with 100nM dexamethasone. Data is shown for 1 hour and 24h with (1h+23w) and without (24h) washout of the ligand. Error bars are 2 x standard error of the mean (SEM) for 3 technical replicates.
